# PIK3CA-related overgrowth with an uncommon phenotype: case report

**DOI:** 10.1186/s13052-022-01268-9

**Published:** 2022-05-12

**Authors:** Roberta Rotunno, Andrea Diociaiuti, Elisa Pisaneschi, Claudia Carnevale, Marialisa Dentici, May El Hachem

**Affiliations:** 1 Dermatology Unit, Bambino Gesù Children’s Hospital-IRCCS, P.zza St. Onofrio 4, 00165 Rome, Italy; 2 VASCERN VASCA and ERN-Skin European Reference Centre, Rome, Italy; 3 Medical Genetics Laboratory, “Bambino Gesù” Children’s Hospital, IRCCS, Rome, Italy; 4 Unit of Medical Genetics, Bambino Gesù Children’s Hospital, IRCCS, Rome, Italy

**Keywords:** PIK3CA, Megalencephaly-capillary malformation, Phenotype

## Abstract

**Background:**

Megalencephaly-capillary malformation syndrome is a rare multiple-malformation syndrome secondary to somatic activating mutations in the PI3K-AKT-MTOR pathway. This is included in a heterogeneous group of disorders, now defined “PIK3CA-related overgrowth spectrum”.

**Case presentation:**

We report a 22-months-old female presenting an uncommon phenotype associated with a genetic mosaicism in the PIK3CA gene, detected on DNA extracted from blood peripheral and tissue biopsy.

**Conclusions:**

NGS is the preferred method for molecular diagnosis of PROS on affected skin and overgrown tissues as primary samples. The wide phenotypic variability is based on the distribution of mosaicism, in fact the same mutation can cause different PIK3CA related disorders. Continuous understanding of the clinical spectrum and of molecular basis of PROS and their overlap will lead to improve diagnosis, management and new treatment strategies.

## Background

Several overgrowth conditions are caused by postzygotic activating mutations in the PIK3CA/AKT/mTOR pathway that occur during embryogenesis and result in a mosaic organism composed of genetically different cell populations. These disorders, now defined “PIK3CA-related overgrowth spectrum” (PROS), present a wide range of phenotypic variability [1, 2]. In this heterogeneous group are included numerous already known and emerging clinical entities, such as Fibroadipose hyperplasia or Overgrowth (FAO), Hemihyperplasia Multiple Lipomatosis (HHML), Congenital Lipomatous Overgrowth, Vascular Malformations, Epidermal Nevi, Scoliosis/Skeletal and Spinal (CLOVES) syndrome, Fibroadipose Infiltrating Lipomatosis, and the related megalencephaly syndromes, Megalencephaly-Capillary Malformation (MCAP or M-CM), Dysplastic Megalencephaly (DMEG), Klippel-Trenaunay syndrome (KTS), and some cases of macrodactyly [2–4]. The wide range of phenotypic overgrowth spectrum and their overlapping features may be explained by the timing of the mutational occurrence during the embryonic development, as well as the destiny of the mutated cell (tissue specificity), the percentage of mosaicism and the potential allelic heterogeneity [5]. Here, we report an Italian female case of PROS presenting an uncommon phenotype associated with a genetic mosaicism in the PIK3CA gene, detected on DNA extracted from blood peripheral and tissue biopsy.

## Case presentation

**Fig. 1:**
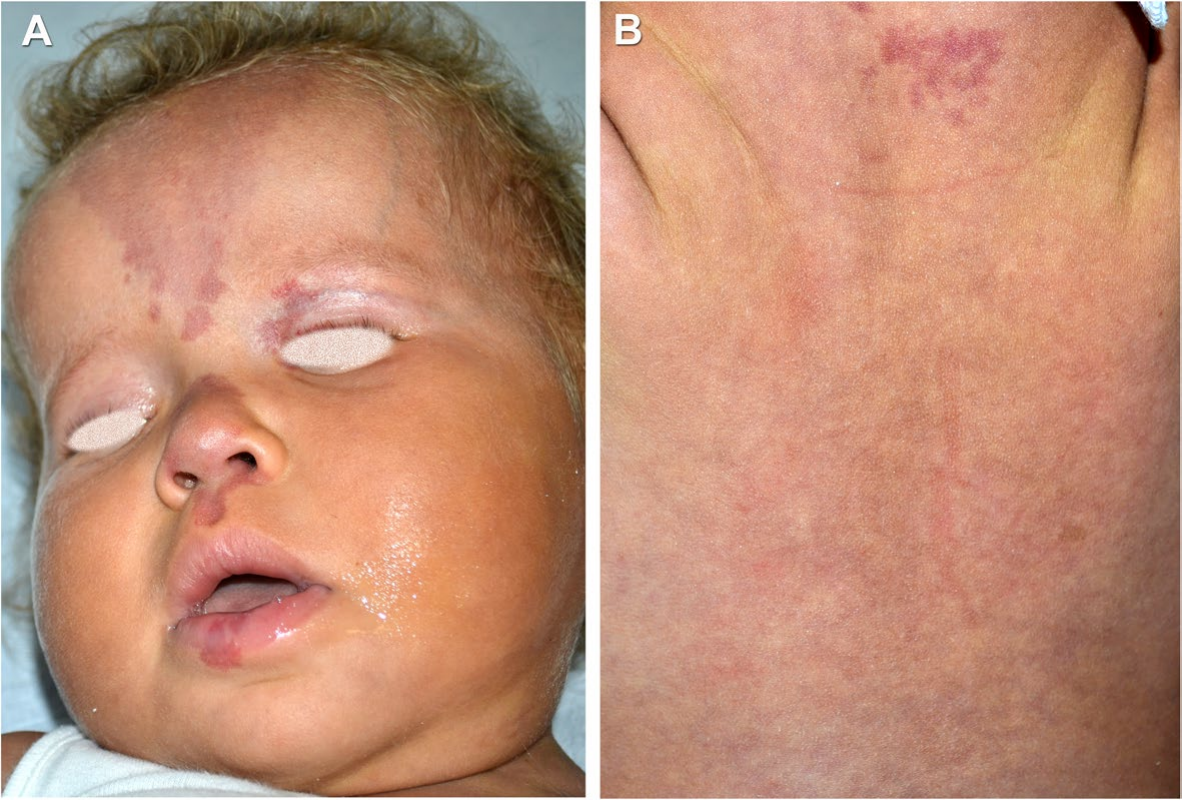
Diffuse reticulated capillary malformations were visible all over the body, more evident on the back and on the face, as a V-shaped stain of the forehead, the upper eyelids, the nose, the filter and the lower lip (**A**, **B**)

**Fig. 2:**
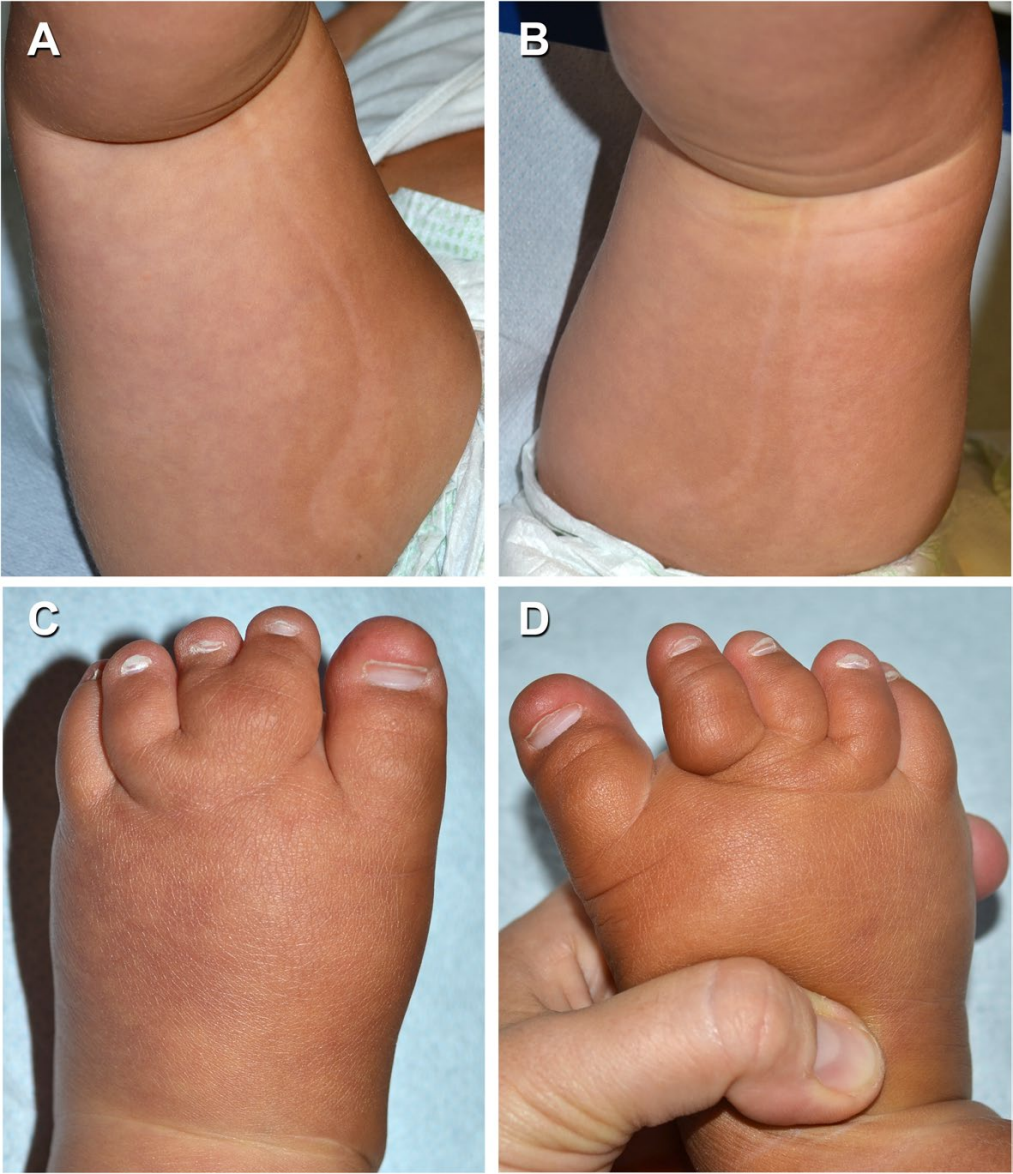
Bilateral linear hypopigmented macules distribuited along the Blaschko lines on the back surface of the thighs (**A**, **B**); syndactyly of II and III toes (**C**, **D**)

**Fig. 3:**
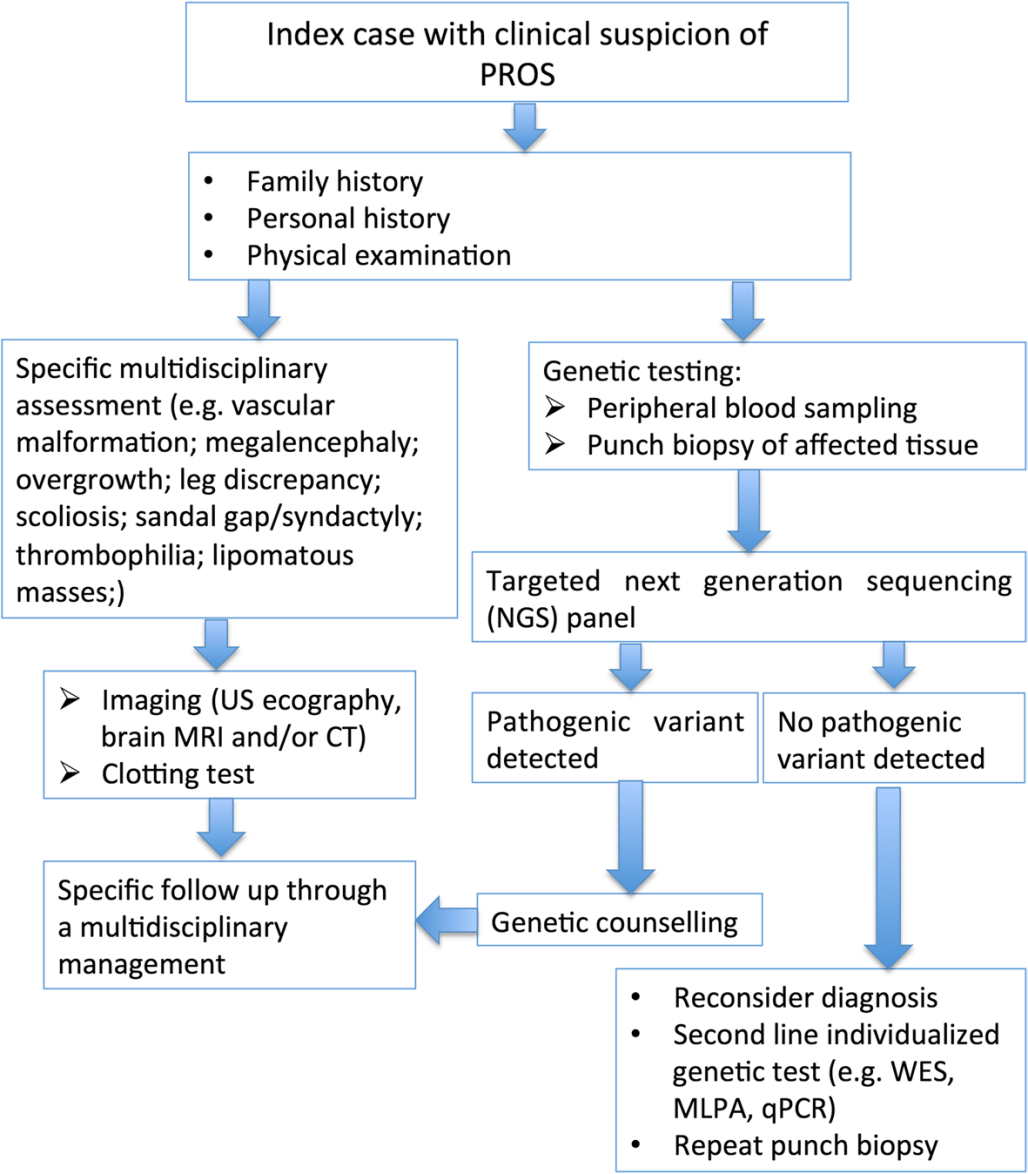
Flow chart - clinical and molecular assessment in the PROS patients

A 22-months-old female was born at 39 weeks of gestational age by cesarean section, for a suspect fetal macrosomia during the pregnancy. At birth weight was 4720 g, length was 54 cm, occipito-frontal circumference was 40.5 cm, and diagnosis of hip dysplasia was made. There was no family history of neurologic disease or developmental delay. During the perinatal period she was hospitalized due to macrosomia and hypotonia. A brain Magnetic Resonance Imaging (MRI) performed in the first weeks of life showed micropoligyria. The patient also underwent physiokinesitherapy to treat evolutionary delay, diffuse articular laxity with instability of the knees. Her hip dysplasia was treated with blunt retractor. A radiography revealed partial agenesis of the vertebral body that corresponded to a swelling at lumbar spine level. A karyotype analysis was performed detecting a paternal pericentric inversion of chromosome 2. On physical examination, macrocephaly and hypotonia were present associated to hyperextensible joints, overgrowth of the lower limbs and syndactyly of II and III toes. Capillary malformations were visible all over the body. They appeared diffuse reticulated on the limbs and the back, and on the medial area of the face, involving the forehead and glabella, as a V-shaped stain, the upper eyelids, the nose, the filter and the lower lip. (Fig. 1 A-B) Bilateral linear hypopigmented macules distributed along the Blaschko lines were observable on the back surface of the thighs. (Fig. 2 A-D) On the basis of these clinical signs, we suspected a PROS disorder, ruling out other overgrowth syndromes (e.g. Sotos, Beckwith-Wiedemann. Pallister-Killian). Thus, we performed molecular analysis with next generation sequencing of selected genes involved in the PI3K/AKT/mTOR pathway on DNA extracted from peripheral blood and from cutaneous biopsy of affected tissue. (Fig. 3) The analysis revealed a pathogenic mosaic mutation c.344G > C in PIK3CA gene with a frequency of the mutated allele of about 12% on DNA extracted from peripheral blood and about 15% on DNA extracted from tissue biopsy. At the protein level it determines the p.Arg115Pro variant. The variant in PIK3CA gene was previously described [4, 6]. The histological findings on punch biopsy specimens showed the presence of small ectatic blood capillaries (CD31+, CD34 +, GLUT1-, D240-) compatible with the clinical diagnosis of capillary malformation. The patient is in follow up and she still presents macrocephaly, macrosomia and hypotonia. Thus a multidisciplinary management, including physiokinesitherapy is ongoing.

## Discussion and conclusion

The PI3K/AKT/mTOR pathway is among the most important signalling pathways in the cell [6, 7]. Mutations in this large cell-signalling axis, first known in cancer, have been linked to megalencephaly and overgrowth syndromes (MCAP, CLOVES, Klippel-Trenaunay or Proteus syndromes) or localized body overgrowth (macrodactyly) [3, 8–10]. These disorders are variably accompanied with other malformations, including musculoskeletal abnormalities (scoliosis, bone overgrowth) and vascular and lymphatic malformations, and single tissue dysplasia (epidermal and other types of skin nevi, lipomatosis) [10, 11]. Because of the phenotypic variability of these disorders caused by somatic PIK3CA mutations, the umbrella-term PROS was proposed to encompass all these clinical entities and highlights the spectrum of findings [2]. In fact, the clinical presentation of the patients is variable, and exists considerable overlap between PROS entities [5]. The finding of a mosaic mutation can be very useful to establish a diagnosis of a PROS disorder. Nevertheless, some mutations appear to have tissue-specific distribution, while others are more pleiotropic; thus the correlation of the mutation to manifestations and disease severity is poor [2, 3, 5, 12]. Based on clinical and instrumental findings, we diagnosed our case of PROS as MCAP, according to the diagnostic criteria proposed by Mirzaa et al. [9, 10] We were also supported by the association, already reported in literature between the phenotype and the pathogenic mutation p.Arg115Pro in PIK3CA gene [13]. In particular, the heterozygous variant R115P in PIK3CA gene found in our patient was originally reported in an 8-year-old girl who presented isolated macrodactyly, with enlarged index and middle fingers on one hand, with other digits being normal and no other vascular abnormalities were noted. The novel mutation was detected in mosaic, present only in the affected nerve tissue in a percentage of about 28%, but not in blood DNA [4]. Subsequently, the same variant was reported in 2 patients affected by CLAPO syndrome in which the mutation was detected on skin biopsy of affected tissue with percentage of mosaicism ranging from 7.5 to 16%. In one of the two patients, the mutation was also found in mosaic at a level of 1.26% in a blood sample [6]. The PIK3CA (R115P) was so far, not reported in patients with a clinical phenotype closer to MCAP. This syndrome is characterized by congenital or early postnatal megalencephaly, with a progressive ventriculomegaly leading to hydrocephalus and cerebellar tonsillar ectopia leading to Chiari malformation, and cortical brain abnormalities, specifically polymicrogyria [9, 10]. MCAP is associated also with other features, such as cutaneous vascular malformations, mainly capillary malformations of the face and reticulated capillary malformation on the trunk; digital anomalies consisting of syndactyly, polydactyly or both; connective tissue dysplasia as joint hypermobility; focal or segmental body overgrowth; hypotonia, and mild to severe intellectual disability [9, 10]. Our patient however presented an uncommon phenotype. In fact, in addition to the most typical features of MCAP, we observed a bilateral hypopigmented, linear skin lesions on her lower limbs. This feature has been described only by Choi et al. in a Korean female diagnosed with MCAP on the basis of clinical and neuroradiological findings, but without confirmation with genetic investigation [14]. Mathew et al. described a case of FAO with blaschkoid hypopigmentation on the neck which was found to be unrelated to the overgrowth [15].

In literature, several clinical reports of hemimegalencephaly and skin pigmentary mosaicism are described, initially so-called hypomelanosis of Ito. Then, Dobyns and Mirzaa designated this condition as the megalencephaly-polymicrogyria-pigmentary mosaicism syndrome (MPPM) [9, 16]. Among these cases, none is caused by PIK3CA mutation, but mosaic mutations of MTOR gene, within the same PI3K/AKT/mTOR pathway, are associated with this syndrome [9, 15]. The skin phenotype related with mutations of MTOR gene corresponds to cutis tricolor of the Blaschko-linear type, a form of pigmentary mosaicism, previously defined “hypomelanosis of Ito” and reflecting the association with hypopigmented and hyperpigmented streaks [12, 16]. The diagnostic criteria for MCAP syndrome have been changed and reported by many clinical groups, therefore describing new cases with different features is important to enrich these criteria [9, 10]. Causative germline or postzygotic mutations in the PIK3CA, PIK3R2 and AKT3 genes have been identified in patients with megalencephaly and polymicrogyria [10]. We detected in our patient PIK3CA mosaic mutation in blood and affected tissue samples with a frequency of the allele mutated to about 12 and 15%, respectively. Literature data showed that blood and buccal samples lead to lower diagnostic rates and mutant allele levels than skin and other affected tissues [11]. We therefore recommend affected skin and overgrown tissues as primary samples for molecular diagnosis of PROS, and blood sample as control. As in our case, NGS is the preferred method for molecular diagnosis of PROS because it offers a much deeper sequencing coverage and allows the detection of low-level mosaicism [11]. The wide phenotypic variability observed, not only in MCAP, but in all the PROS is not surprising, given the mosaic nature and the genetic heterogeneity of these syndromes. Indeed, we expect that the phenotype may differ based on distribution of mosaicism. Moreover, our patient provides additional evidence that a rigid nosological distinction between the different PIK3CA-associated segmental overgrowth entities cannot longer be applied. The same mutation can be found in extreme phenotypes of the PIK3CA related disorders, ranging from isolated macrodactyly, to CLOVE syndrome, to MCAP (as in our patient) with a variable percentage in tissue lesions and, in some cases, in blood. Our findings provide evidence for a wide phenotypic diversity associated with the same mutation affecting PIK3CA, and occurrence of a clinical continuum associated with PIK3CA molecular defects. Continuous understanding of the clinical spectrum and of molecular basis of PROS and their overlap will lead to improve diagnosis. In particular, we need to further define the phenotypic spectrum of germline and somatic mutations in PIK3CA, with the aim of paving the way to improved management and new treatment strategies.

## Data Availability

Data sharing is not applicable to this article as no datasets were generated or analysed during the current study.

## Abbreviations

PROS: PIK3CA-related overgrowth spectrum
FAO: Fibroadipose hyperplasia
HHML: Overgrowth Hemihyperplasia Multiple Lipomatosis
CLOVES: Congenital Lipomatous Overgrowth, Vascular Malformations, Epidermal Nevi, Scoliosis/Skeletal and Spinal syndrome
MCAP: Fibroadipose Infiltrating Lipomatosis, and the related megalencephaly syndromes,
M-CM: Megalencephaly-Capillary Malformation
DMEG: Dysplastic Megalencephaly
KTS: Klippel-Trenaunay syndrome
MRI: Magnetic Resonance Imaging
CLAPO: Capillary vascular malformation of the lower lip, Lymphatic malformations of the head and neck, Asymmetry and Partial or generalized Overgrowth
